# The efficacy of corticosteroid after facial nerve neurorrhaphy: a systematic review and meta-analysis of randomized controlled trial

**DOI:** 10.1016/j.bjorl.2021.09.005

**Published:** 2021-11-02

**Authors:** Prapitphan Charoenlux, Nattawan Utoomprurkporn, Kachorn Seresirikachorn

**Affiliations:** a Department of Otolaryngology, Faculty of Medicine, Chulalongkorn University, Bangkok, Thailand; b UCL Ear Institute, Faculty of Brain Science, University College London, London, United Kingdom; c Endoscopic Nasal and Sinus Surgery Excellence Center, King Chulalongkorn Memorial Hospital, Bangkok, Thailand

**Keywords:** Corticosteroid, Facial nerve neurorrhaphy, Complete facial nerve transection, Nerve regeneration, Functional recovery

## Abstract

**Objectives:**

The benefit of corticosteroids following facial nerve neurorrhaphy in the setting of complete transection is questionable. This systematic review and meta-analysis aimed to evaluate corticosteroid efficacy on facial nerve regeneration and functional recovery after complete disruption and neurorrhaphy.

**Methods:**

Randomized controlled trials on both human and animal models from Ovid MEDLINE and Ovid EMBASE studying corticosteroid efficacy in complete facial nerve disruption followed by neurorrhaphy were included. Data were extracted and pooled for meta-analysis. The outcomes were evaluated from electrophysiology, histology, and functional recovery. However, no randomized controlled trial in human was performed. Possibly, performing human trials with histopathology may not be feasible in clinical setting.

**Results:**

Six animal studies (248 participants) met inclusion criteria. Electrophysiologic outcomes revealed no differences in latency (Standardized Mean Difference (SMD) = −1.97, 95% CI −7.38 to 3.44, *p* = 0.47) and amplitude (SMD = 0.37, 95% CI −0.44 to 1.18, *p* = 0.37) between systemic corticosteroids and controls. When analysis compared topical corticosteroid and control, the results provided no differences in latency (Mean Difference (MD) = 0.10, 95% CI −0.04 to 0.24, *p* = 0.16) and amplitude (SMD = 0.01, 95% CI −0.08 to 0.10, *p* = 0.81). In histologic outcomes, the results showed no differences in axon diameter (MD = 0.13, 95% CI −0.15 to 0.41, *p* = 0.37) between systemic corticosteroid and control; however, the result in myelin thickness (MD = 0.06, 95% CI 0.04 to 0.08, *p* < 0.05) favored control group. When comparing systemic corticosteroid with control in eye blinking, the results favored control (MD = 1.33, 95% CI 0.60 to 2.06, *p* =  0.0004).

**Conclusions:**

This evidence did not show potential benefits of systemic or topical corticosteroid deliveries after facial nerve neurorrhaphy in complete transection when evaluating electrophysiologic, histologic, and functional recovery outcomes in animal models.

## Highlights

• The benefits of corticosteroid after facial nerve neurorrhaphy are questionable.
• Corticosteroid was not provided benefits after coaptation in complete transection.
• The benefits were judged by electrophysiology, histology, and functional recovery.

## Introduction

The facial nerve is a mixed nerve consisting of motor, sensory and parasympathetic fibers. It can be classified by its anatomical location as intracranial, intratemporal, and extratemporal parts where the more distal part has more motor fibers.^1^ Complete axonal disruption of the facial nerve results from various etiologies, for example, traumatic facial nerve injury, iatrogenic injury in the parotid, soft tissue, orthognathic, or otologic surgery, and oncologic control surgery of head-and-neck cancer.^1^ Total facial nerve paralysis may cause facial asymmetry, corneal ulcer, inability to elevate the forehead, midface ptosis, unnatural or inability to smile which may lead to patient morbidity.^2^

After nerve injury, an inflammatory reaction occurs with macrophage function at the distal peripheral nerve site, followed by Wallerian degeneration and demyelination. Subsequently, the nerve regeneration process begins.^3^ Molecular mechanisms of peripheral nerve repair are remyelination, axonal sprouting, and axonal regeneration.^4^ After the injury, Schwann cells begin to divide and proliferate. In the final stage, the axons enter the endoneurial tube of the distal stump.^3^ Neuroinflammation is the main process after nerve injury, therefore, corticosteroids that reduce perineural inflammation in many diseases are used in facial nerve injury.

Mechanisms of corticosteroids in nerve injury include (1) reduce neural edema and perineural inflammation, (2) protect cells from peroxidation, (3) prevent motor neuron death, (4) lower anterograde degeneration rate, and (5) promote recovery.^5^, ^6^, ^7^, ^8^, ^9^ In partial injury of the facial nerve, corticosteroid is shown to improve facial nerve regeneration with a higher recovery rate.^10^, ^11^, ^12^, ^13^, ^14^ Consequently, clinical trials and guidelines recommend high dose corticosteroids for partial facial nerve injury.^10^, ^15^, ^16^ In contrast, the gold standard treatment of complete nerve injury is an immediate tension-free neurorrhaphy with end-to-end anastomosis or nerve interposition graft for the best functional outcome.^17^, ^18^ However, functional recovery does not achieve the pre-injury level. Adjunctive therapy with corticosteroids after facial nerve coaptation is proposed and prescribed in a general clinical setting.

Nevertheless, the clinical benefit is questionable, and there is no high level of evidence to support the use of corticosteroids. Moreover, there are risks associated with corticosteroid usage. Adverse effects, including gastrointestinal disturbance, increased blood glucose level, elevated blood pressure, and psychotic episodes have been reported.^19^, ^20^ As a consequence, corticosteroid usage should be investigated to confirm its clinical benefits. Thus, this systematic review and meta-analysis aimed to evaluate corticosteroid efficacy on facial nerve regeneration and functional recovery after neurorrhaphy in the setting of complete axonal disruption.

## Methods

### Eligibility criteria

This systematic review followed the guidelines provided by the Preferred Reporting Items for Systematic reviews and Meta-Analyses (PRISMA) statement.^21^ Randomized Controlled Trials (RCTs) studying the efficacy of corticosteroid therapy after microsuture repair, in subjects with complete disruption of the extratemporal facial nerve, were screened. The microsuture repair included direct end-to-end anastomosis or nerve interposition graft. Studies of either human or animal models were eligible. Corticosteroid administrations at any route, dosage, frequency, and duration after facial nerve coaptation were included in the analysis. The comparisons were (1) systemic corticosteroid versus no corticosteroid (control), (2) local corticosteroid versus no corticosteroid (control), and (3) systemic corticosteroid versus local corticosteroid. Exclusion criteria included RCTs that were published in a language other than English.

### Information sources and search strategy

Ovid MEDLINE and Ovid EMBASE were searched using the search terms: "Dexamethasone OR Methylprednisolone OR Prednisolone OR Corticosteroids OR Triamcinolone OR Steroids OR Hydrocortisone OR Glucocorticoids" AND "Neurorrhaphy OR Nerve anastomosis OR Nerve suture OR Suturing method OR Suturing technique OR End-to-end anastomosis OR End-to-end method OR Nerve graft OR Nerve autograft OR Nerve interposition graft OR Microsuture OR Microsurgery OR Nerve surgery OR Nerve coaptation" AND "Facial nerve". The last search was performed on 20 April 2021. References of the included studies were searched for identifying any missing published or unpublished trials.

### Study selection and data collection

The RCTs selection was performed independently by two reviewers (PC and KS). The reviewers independently screened the titles and abstracts based on the predetermined eligibility criteria. Full texts of the selected articles were reviewed. Any disagreements were resolved by another author (NU), if necessary. Two review authors (PC and KS) independently extracted data from the included studies using a predetermined data collection form. The extracted data included study type, number of participants, animal type, age, sex, intervention, primary outcomes, and secondary outcomes. If there were many doses of corticosteroids in one study, the recommended high dose of 50–60 milligrams per day (which was equivalent to 1 mg/kg/dose) from the guideline^10^ was extracted for the meta-analysis. Primary outcomes were two aspects of nerve regeneration, which included (1) electrophysiology which assessed the latency and amplitude values of electroneurography, and (2) histology which evaluated axon diameter and myelin thickness. Secondary outcomes were functional recovery evaluated from eye blinking and adverse events. Standard error, interquartile range, and 95% Confidence Interval (95% CI) were used when a Standard Deviation (SD) was not reported.

### Risk of bias in individual studies

Risk of bias of the included studies was assessed according to the Cochrane Handbook for Systematic Reviews of Interventions. Five domains were evaluated: random sequence generation, allocation concealment, blinding of participants and personnel, incomplete outcome data, and selective reporting.^22^ The included studies had low risk of bias when the methods for each domain were clearly described. When the described methods for each domain showed a high risk of bias, that study was classified as high risk in that domain. When there was not enough information to determine the risk, the RCT was defined as unclear risk of bias.

### Data synthesis and statistical analysis

Data were pooled for meta-analysis. Odds Ratio (OR) and 95% CI were used for dichotomous data. Mean Difference (MD), Standard Mean Difference (SMD), and 95% CI were used for continuous data. Heterogeneity or discrepancy in the estimates of treatment effects from different trials were assessed by I^2^ statistic. An I^2^ of less than 40%, 40%–60%, or >60% represented low, moderate, and substantial heterogeneity, respectively. A fixed-effect method was used when the statistical heterogeneity was low. When the statistical heterogeneity was high, a random-effect method was used for a more conservative estimate of the difference. Statistical assessment was performed with Review Manager (RevMan) version 5.4 (The Nordic Cochrane Center, The Cochrane Collaboration, Copenhagen, Denmark).^23^, ^24^, ^25^

## Results

### Study selection

**Figure 1:**
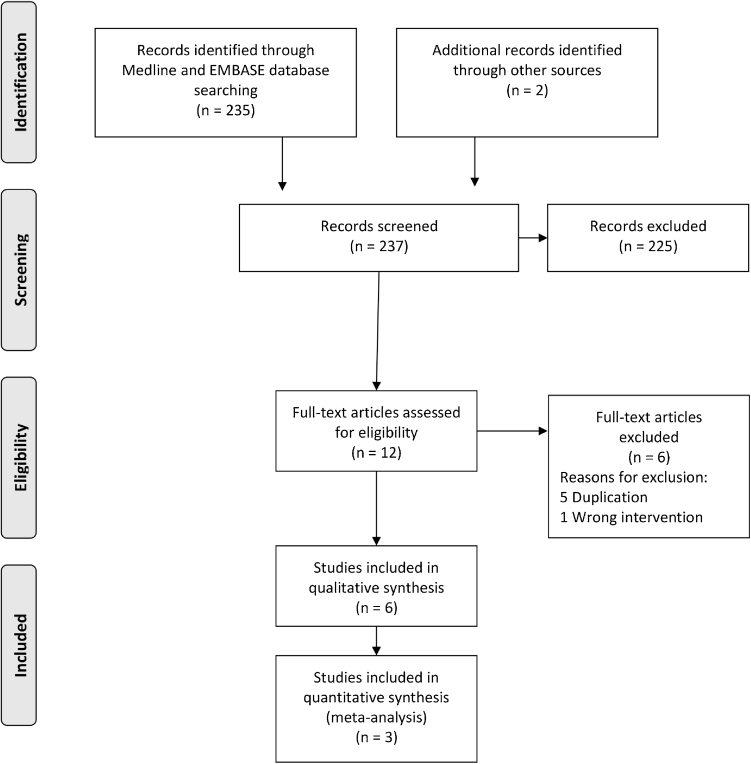
A flow chart of the study retrieval and selection: The PRISMA flowchart of the systematic literature reviews.

There were 237 studies identified and retrieved, of which 235 were from electronic searches, and two from manual searches. During the title and abstract screening, 225 studies were excluded due to irrelevant references. Six studies were excluded after the full-text screening. Six studies^26^, ^27^, ^28^, ^29^, ^30^, ^31^ were finally included in the qualitative synthesis, of which three studies^26^, ^27^, ^28^ were included in the meta-analysis. Characteristics of the included studies are shown in Table 1. A flow chart of the study retrieval and selection is presented in Fig. 1.

**Table 1:** Characteristic data.

Nº	Author	Year	Animal	N	Site of FN	Type of intervention	Materials & methods	Time	Groups	Dose	Freq.	Dur.	Route	N per group
1	Karlidag et al.^31^	2011	New Zealand rabbit	30	Buccal branch	Complete transection with E-t-E anastomosis	Prolene 9–0, epineural suture	8 wks.	Control	Received no medication				10
									*N*-Acetylcystein	50 mg/kg	OD	2 mo.	IM	10
									Methylprednisolone	1 mg/kg	OD	2 mo.	N/A	10
2	Seth et al.^27^	2012	Wistar rat	74	Left main trunk	Complete transection with tension-free microsuture coaptation	Nylon 10–0, perineural suture	8 wks.	Control (saline)	N/A	N/A	N/A	Local + IP	12
									Systemic dexamethasone (+gelfoam saline)	1 mg/kg	12 h apart	3-times	IP	12
									Systemic dexamethasone (+gelfoam saline)	5 mg/kg	12 h apart	3-times	IP	12
									Local dexamethasone (+inject saline)	2 mg/mL	1-time Intraop	1-time	Local	12
									Local dexamethasone (+inject saline)	4 mg/mL	1-time Intraop	1-time	Local	12
									Systemic dexamethasone (+gelfoam saline)	0.5 mg/kg	12 h apart	3-times	IP	7
									Systemic dexamethasone (+gelfoam saline)	10 mg/kg	12 h apart	3-times	IP	7
3	Yildirim et al.^30^	2014	New Zealand rabbit	30	Buccal branch	Nerve transection	Ethicon 9–0 epineural	8 wks.	Methylprednisolone	1 mg/kg	OD	3 wks.	IM	5
									Control	1 mL NSS	OD	3 wks.	IM	5
						Nerve compression	No repair		Methylprednisolone	1 mg/kg	OD	3 wks.	IM	5
									Control	1 mL NSS	OD	3 wks.	IM	5
						HSV type 1 inoculation			Methylprednisolone	1 mg/kg	OD	3 wks.	IM	5
									Control	1 mL NSS	OD	3 wks.	IM	5
4	Yanilmaz et al.^29^	2014	New Zealand rabbit	32	Left buccal branch	Complete transection with E-t-E anastomosis	Prolene 9–0 epineural repair	10 wks.	Control	No medication				8
									Aminoguanidine	100 mg/kg	OD	14d.	IP	8
									Melatonin	30 mg/kg	OD	10d.	IP	8
									Methylprednisolone	1 mg/kg	OD	15–18d.	IM	8
5	Edizer et al.^26^	2018	Albino Wistar rat	50	Left main trunk	Complete transection with E-t-E anastomosis	Nylon 8–0, epineural suture	13 wks.^a^	Control (saline)	N/A	N/A	7d.	Local + IP	10
									Topical melatonin (+IP saline)	Conc. 20 mg/mL	1-time	1-time	Local	10
									Systemic melatonin (+ topical saline)	20 mg/kg	OD	7d.	IP	10
									Topical dexamethasone (+ IP saline)	Conc. 4 mg/mL	1-time	1-time	Local	10
									Systemic dexamethasone (+ topical saline)	1 mg/kg	OD	7d.	IP	10
6	Longur et al.^28^	2020	Wistar rat	32	Right main trunk	Full-thickness cut with E-t-E anastomosis	Prolene 8–0, mattress suture	28d.	Control	Received no intervention				8
									Bumetanide	15 mg/kg	OD	7d.	Gav.	8
									Dexamethasone	1 mg/kg	OD	7d.	IP	8
									Bumetanide + Dexamethasone	15 mg/kg +1 mg/kg	OD	7d.	Gav. + IP	8

Conc., Concentration; d., day(s); Dur, duration; E-t-E, end-to-end, FN, facial nerve, Freq., frequency; Gav., gavage, h, hours; IM, intramuscular; Intraop, intraoperation; IP, intraperitoneal; kg, kilogram; mg, milligram; mL, milliliter; mo., month(s); N/A, not available data; NSS, normal saline; N, number; OD, once daily; wk.(s), week(s).

a Total study period was 13-weeks (drug was administrated for 7 consecutive days after the operation. The analysis at the end of the study was performed 12-weeks after the end of drug delivery).

### Participants

There were no human studies that met the inclusion criteria. Three studies were rat models, and the others were New Zealand rabbit models. There were 248 animals, of which 156 were Wistar rats (106 Wistar rats,^27^, ^28^ 50 Albino Wistar rats^26^), and 92 were New Zealand rabbits.^29^, ^30^, ^31^ In the Wistar rat models, 124 animals were male,^26^, ^27^ and 32 animals were unidentified sex.^28^ The rats weighed from 200 to 350 g. The age was 12–14 weeks old in one study^28^ but was not mentioned in the other two studies.^26^, ^27^ In the New Zealand rabbit models, 60 animals were female^30^, ^31^ and 32 animals were unidentified sex.^29^ The rabbits weighed from 1200 to 3000 grams. The age was not discussed in all rabbit model studies.

### Interventions

The animals were housed at 21–25 °C under 10%–55% humidity. They were exposed to a 12-hour light/dark cycle and were fed with a standard diet. General anesthesia was induced with a combination of Ketamine Hydrochloride (30–50 mg/kg) and Xylazine Hydrochloride (5–10 mg/kg) intraperitoneally. All included studies performed a completed transection of the facial nerve at one side and the other side was used as control. All animals exhibited postoperative facial paralysis. An immediate repair with tension-free, end-to-end microsuture coaptation was performed with Prolene 8–0 in one study,^28^ Nylon 8–0 in one study,^26^ Prolene 9–0 in three studies,^29^, ^30^, ^31^ and Nylon 10–0 in the remaining study.^27^ Epineural repair technique was used in four studies,^26^, ^29^, ^30^, ^31^ perineural suture was used in one study.^27^ However, one study did not mention the repair technique.^28^

Systemic corticosteroid was given in six studies,^26^, ^27^, ^28^, ^29^, ^30^, ^31^ and topical form was applied in two studies.^26^, ^27^ Systemic dexamethasone was administered daily at 1 mg/kg/dose via intraperitoneal injection for seven consecutive days in two studies^26^, ^28^ and one study administered a total of three consecutive doses, 12 hour apart.^27^ Systemic methylprednisolone (1 mg/kg/dose) was injected intramuscularly once daily for 15–18 days in one study,^29^ three weeks in one study,^31^ and two months in one study.^30^ Topical administration of dexamethasone-soaked gelfoam in 4 mg/mL concentration was applied intraoperatively in two studies.^26^, ^27^ The studied period ranged from 4 to 13 weeks.^26^, ^27^, ^28^, ^29^, ^30^, ^31^ The preoperative baseline of electrophysiologic evaluation with electroneurography was evaluated on both sides.

### Outcomes

#### Electrophysiologic results

The electrophysiologic outcomes were assessed by nerve conduction test using Neuro-MEP 2 channel digital instrument at 10%–20% supramaximal intensity. Latency (millisecond) and amplitude (millivolts) were recorded.

**Figure 2:**
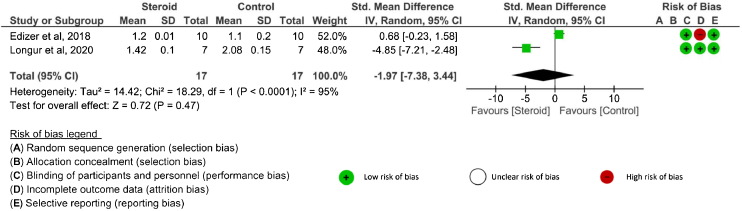
The latency outcome of systemic corticosteroid at the end of study. Abbreviations: SMD, Standardized Mean Difference; IV, Inverse Variance; Random, Random effects; CI, Confidence Interval.

*Latency value:* There were three^26^, ^28^, ^29^ studies that evaluated latency outcomes. However, one study^29^ did not report either SD or 95% CI. Two studies^26^, ^28^ reported latency after systemic corticosteroid administration and one RCT^26^ evaluated latency after topical application. The latency was assessed at 4-weeks, ≥12 weeks, and at the end of the study in the systemic route. There were no significant differences in latency between the corticosteroids and control groups at any time point: 4 weeks (SMD = −2.58, 95% CI −6.73 to 1.57, *p* = 0.22, 2 RCTs),^26^, ^28^ ≥12-weeks (MD = 0.10, 95% CI −0.02 to 0.22, *p* = 0.11, 1 RCT),^26^ and the end of the study (SMD = −1.97, 95% CI −7.38 to 3.44, *p* = 0.47, 2 RCTs)^26^, ^28^ (Fig. 2). An I^2^ of 91% (4 weeks) and 95% (the end of study) represented substantial heterogeneity. In the topical corticosteroid application, there was no significant difference in latency between the topical steroid and control groups at the end of the study (MD = 0.10, 95% CI −0.04 to 0.24, *p* = 0.16, 1 RCT).^26^ When topical corticosteroid administration was compared with systemic corticosteroid administration, there was no significant difference in latency at the end of the study (MD = 0.00, 95% CI −0.06 to 0.06, *p* =  1.00, 1 RCT).^26^

**Figure 3:**
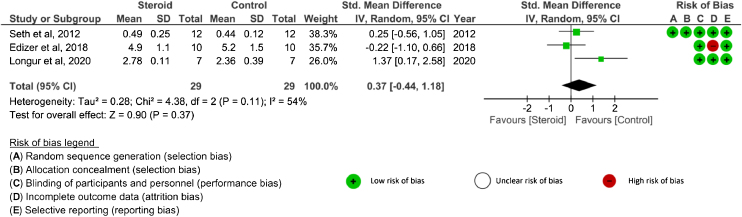
The amplitude outcome of systemic corticosteroid at the end of study. Abbreviations: SMD, Standardized Mean Difference; IV, Inverse Variance; Random, Random effects; CI, Confidence Interval.

**Figure 4:**
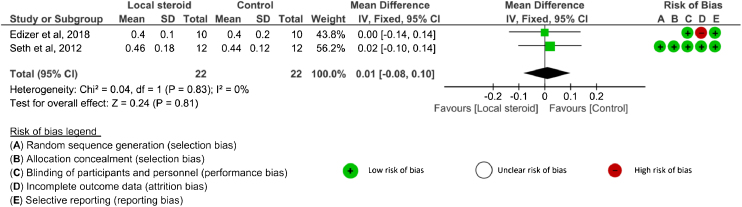
The amplitude outcome of topical corticosteroid at the end of study. Abbreviations: SMD, Standardized Mean Difference; IV, Inverse Variance; Random, Random effects; CI, Confidence Interval.

Amplitude value: Four studies^26^, ^27^, ^28^, ^29^ analyzed amplitude outcomes. SDs or 95% CIs were not reported in one study.^29^ Three studies^26^, ^27^, ^28^ evaluated amplitude after systemic corticosteroid administration and two studies^26^, ^27^ analyzed amplitude after topical application. The amplitude was assessed at 4 weeks, 8 weeks, ≥ 12 weeks, and the end of the study in the systemic group.^26^, ^28^ There were no significant differences in amplitude between the corticosteroid and control groups at any time point: 4 weeks (SMD = 0.49, 95% CI −1.13 to 2.11, *p* = 0.55, 2 RCTs), 8 weeks (MD = 0.05, 95% CI −0.11 to 0.21, *p* = 0.53, 1 RCT), ≥ 12 weeks (MD = −0.30, 95% CI −1.45 to 0.85, *p* = 0.61, 1 RCT), and the end of study (SMD = 0.37, 95% CI −0.44 to 1.18, *p* = 0.37, 3 RCTs)^26^, ^27^, ^28^ (Fig. 3). An I^2^ of 79% represented substantial heterogeneity at 4 weeks, and I^2^ of 54% represented moderate heterogeneity at the end of the study. In the analysis of local corticosteroid application, there was no significant difference in amplitude between the corticosteroid and control groups at the end of the study (SMD = 0.01, 95% CI −0.08 to 0.10, *p* = 0.81, 2 RCTs)^26^, ^27^ (Fig. 4). An I^2^ of 0% represented low heterogeneity. There was no significant difference in amplitude between topical corticosteroid and systemic corticosteroid administrations at the end of the study (SMD = 0.17, 95% CI −0.41 to 0.74, *p* = 0.57, 2RCTs).^26^, ^27^ An I^2^ of 58% represented moderate heterogeneity.

#### Histologic results

The coapted segment of facial nerve was taken, fixed in 2.5% glutaraldehyde and 1% osmium tetroxide, and examined under Transmission Electron Microscope (TEM). The axon diameter and myelin thickness were measured quantitatively. One RCT,^26^ which studied both systemic and topical corticosteroids, assessed axon diameter and myelin thickness outcomes.

*Axon diameter*: One RCT^26^ assessed axon diameter at the end of the study. There was no significant difference in axon diameter between the systemic corticosteroid and control groups (MD = 0.13, 95% CI −0.15 to 0.41, *p* = 0.37, 1 RCT).^26^ When the topical group was compared with the control group, the result favoured the control group (MD = 0.32, 95% CI 0.03–0.61, *p* = 0.03, 1 RCT).^26^ When the topical group was compared with the systemic group, there was no significant difference in axon diameter at the end of the study (MD = 0.19, 95% CI −0.02 to 0.40, *p* = 0.07, 1 RCT).^26^

*Myelin thickness*: One RCT^26^ assessed myelin thickness at the end of the study. When the systemic steroid group was compared with control, the meta-analysis favored control (MD = 0.06, 95% CI 0.04 to 0.08, *p* < 0.05, 1 RCT).^26^ When topical steroid application was compared with control, the result favoured control (MD = 0.04, 95% CI 0.02 to 0.06, *p* = 0.0005, 1 RCT).^26^ When topical steroid group was compared with the systemic corticosteroid group, the result favoured topical route (MD = −0.02, 95% CI −0.04 to −0.00, *p* = 0.03, 1 RCT).^26^

#### Functional results

The degree of eye blinking was graded using a standardized scale. One study^27^ reported the eye blinking result.

*Eye blinking function*: One RCT^27^ assessed eye blinking at the end of the study. When systemic steroid administration was compared with control, the meta-analysis favored control (MD = 1.33, 95% CI 0.60 to 2.06, *p* = 0.0004, 1 RCT).^27^ When topical steroid application was compared with control, the result favoured control (MD = 0.09, 95% CI −0.54 to 0.72, *p* = 0.78, 1 RCT).^27^ When the topical corticosteroid group was compared with the systemic group, the result favoured topical route (MD = −1.24, 95% CI −2.05 to −0.43, *p* = 0.03, 1 RCT).^27^

### Adverse events

In one study,^28^ one animal in the steroid group died in the third week after the operation. However, the cause of death was not clarified. Adverse events in other studies were not reported.

### Risk of bias in the included studies

**Figure 5:**
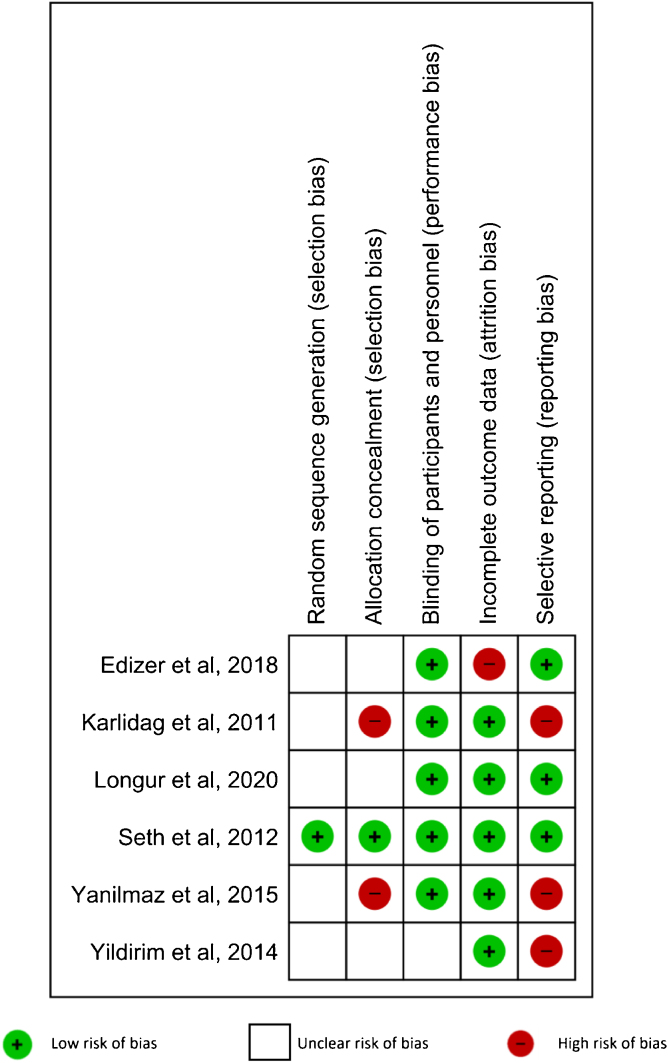
Risk of bias summary: review authors' judgments about each risk of bias item for each included study.

One study^27^ (16.67%) had low risks of bias in randomization and allocation concealment. Five studies^26^, ^27^, ^28^, ^29^, ^31^ (83.33%) had a low risk of bias in blinding participants and personnel. Five RCTs^27^, ^28^, ^29^, ^30^, ^31^ (83.33%) were classified as low risk of bias in incomplete outcome data. Finally, three RCTs^26^, ^27^, ^28^ (50%) had a low risk of bias in selective reporting. The results are shown in Fig. 5.

## Discussion

The results of this systematic review and meta-analysis demonstrated that there were no benefits of systemic or topical corticosteroids in facial nerve regeneration after neurorrhaphy following complete transection in animal models. The nerve regeneration was evaluated by assessing electrophysiologic, histologic, and functional outcomes. In the electrophysiologic outcomes, there were no statistically significant differences in latency or amplitude values: (1) between corticosteroids (both systemic and topical routes) and control, nor (2) between systemic route and topical route. In the histologic and functional outcomes, neither systemic nor topical corticosteroids demonstrated superiority over control in axon diameter, myelin thickness, and eye blinking function.

The meta-analysis favored topical application over systemic corticosteroids in myelin thickness and eye blinking function outcomes. This finding might result from the fact that topical corticosteroid directly contacted the injury site and was absorbed instantly. However, there was only one RCT in the analysis of each outcome.^26^, ^27^ Additional studies should be enrolled for further confirmation.

The meta-analysis result was in line with a previous study by Kardilag et al.^31^ that was not included in the meta-analysis. They reported that methylprednisolone not only had no statistically significant effects on nerve healing after facial nerve re-anastomosis but also further increased degeneration by causing fibrosis in the endoneurium. Furthermore, regeneration (Schwann cell proliferation) was less in the methylprednisolone group compared with the control group and had no superior advantage of steroid over control in myelin thickness. The reason might be collagen scar formation that deteriorated the nerve regeneration.

According to a study by Yanilmaz et al.,^29^ the degree of axonal degeneration and myelin debris accumulation was higher in the corticosteroid group than the control group. Moreover, Schwann cell proliferation was also worse in the steroid group. Yildirim et al.^30^ demonstrated no beneficial effects of methylprednisolone over control in a histologic outcome such as Schwann cell proliferation. In summary, these studies^29^, ^30^, ^31^ showed that corticosteroids had no advantages on nerve regeneration after complete disruption of the facial nerve, including nerve healing,^31^ Schwann cell proliferation,^29^, ^30^, ^31^ or myelin thickness.^31^ Furthermore, corticosteroids might increase the facial nerve degeneration.^29^, ^31^

Neuroinflammation is the first mechanism of neural injury response. It involves several neuroinflammatory cytokines and local production of reactive oxygen species.^32^ Irreversible oxidation caused by free radical leads to cell apoptosis.^33^ The next stage after the neuroinflammatory response are Wallerian degeneration and demyelination. The final stage is regeneration. In the regeneration process, Schwann cells, which are the essential cells involved in remyelination and improving conduction velocity,^3^ begin to divide, proliferate, and guide the regenerated axons to enter the endoneurial tube at the distal stump.^3^ However, this process does not warrant full functional recovery. Complete nerve maturation which involves remyelination, axonal enlargement, and end-organ connection is essential in functional recovery. Moreover, fibrosis and neuroma formation in any steps after nerve injury cause conductive blocking and disruption of the regeneration process. In general, the repair process occurs almost immediately, and regeneration must occur within 12–18 months after the onset of injury before the muscles become atrophied and are replaced with fat or fibrosis.^4^, ^5^, ^6^, ^14^ Although neurorrhaphy was performed immediately after complete facial nerve disruption, the functional recovery in this meta-analysis did not achieve the pre-injury level.

The exact mechanism of why corticosteroid is beneficial in partial nerve injury but not in complete axotomy is still unknown. However, it is believed that corticosteroids reduce the neuroinflammatory process to reduce neural and perineural inflammation and prevent the neurons from perioxidation.^5^, ^6^, ^7^, ^8^, ^9^ The neural injury in neurotmesis is more severe and the repair process is also more complex than those of the partial nerve injury. Perhaps that is why the benefits of corticosteroids could not be achieved. An exact reason needs to be further investigated.

For clinical application in humans, the results of this systematic review and meta-analysis suggested that using corticosteroids, either systemic or topical form, in complete facial nerve disruption and followed by neurorrhaphy should be considered carefully because no benefits were demonstrated. In addition, there are possible side effects of high dose corticosteroids such as increase blood glucose level, glaucoma or cataracts, gastrointestinal irritation, even though they were not examined directly in this systematic review. Therefore, corticosteroid usage should be considered cautiously and balanced between its risks and benefits.

There were limitations in this study: no studies in humans were included in this systematic review. Clinical application in humans is still based on clinical judgement of the clinician for each patient on an individual basis. RCTs in humans should be encouraged in the future for more accurate results. However, performing human trials with histopathology may not be feasible in clinical practice. Without the results in humans, this systematic review could only be used as guidance in clinical practice. The other limitation was only a few RCTs were included in the quantitative assessment in this meta-analysis. If there are more RCTs recruited with more pooled data, this topic could be re-analyzed in the future.

## Conclusion

Evidence from this systemic review and meta-analysis did not show potential benefits of systemic or topical corticosteroid administrations after facial nerve neurorrhaphy in the setting of complete transection. The benefits were evaluated by electrophysiologic, histologic, and functional recovery outcomes in animal models. All available recruited studies did not include human participants, possibly due to the limitations in histologic outcome measurement. However, this study should be a reminder for clinicians in considering corticosteroid usage in such situation and, if possible, prospective human clinical trials are suggested for further study.

## Submission declaration and verification

The work has not been published previously and has not been under consideration for publication elsewhere.

## Ethics committee consideration

This protocol was waived from ethical committee consideration involving human beings or animal due to being review article (systematic review and meta-analysis). No patient consent form was needed in this review.

## Conflicts of interest

The authors declare no conflicts of interest.

## CRediT authorship contribution statement

**Prapitphan Charoenlux:** Conceptualization, Data curation, Formal analysis, Investigation, Methodology, Project administration, Resources, Software, Supervision, Validation, Visualization, Writing – original draft, Writing – review & editing. **Nattawan Utoomprurkporn:** Conceptualization, Data curation, Methodology, Validation, Visualization, Writing – review & editing. **Kachorn Seresirikachorn:** Conceptualization, Data curation, Formal analysis, Investigation, Methodology, Project administration, Resources, Software, Supervision, Validation, Visualization, Writing – review & editing.
